# Novaluron Causes Reduced Egg Hatch After Treating Adult Codling Moths, *Cydia pomenella*: Support for Transovarial Transfer

**DOI:** 10.1673/031.011.12601

**Published:** 2011-09-22

**Authors:** Soo-Hoon S. Kim, John C. Wise, Avhan Gökçe, Mark E. Whalon

**Affiliations:** ^1^ Department of Entomology, Michigan State University, East Lansing, Ml 48824; ^2^ Department of Plant Protection, Gaziosmanpasa University, Tasliciftlik/Tokat, Turkey; ^3^ Department of Entomology, University of Arkansas, 310 Agriculture, Fayetteville, AR 72701

**Keywords:** chitin synthesis inhibitor, horizontal transfer, transovarial transfer

## Abstract

The codling moth, *Cydia pomonella* (L.) (Lepidoptera: Tortricidae), is a primary pest of apples throughout the United States. Reliance on broad spectrum organophosphates has been declining with the slated cancellation and has shifted towards narrow spectrum insecticides. Novaluron, a chitin synthesis inhibitor, has primarily been used for its ovicidal and larvacidal activities. However, recent studies have demonstrated a transovarial effect after exposure to adults. The effects of novaluron were studied to determine if reduced egg hatch occurs after exposure of different sexes to this compound. Effects of this compound through horizontal transfer were also compared with a topical application to *C. pomonella* eggs. Results from independent exposure of different sexes to novaluron were different than the control for all three exposure types; male only, female only, and both treated. The horizontal transfer experiment yielded no significant difference while the topical application of novaluron on eggs showed significantly lower egg hatch. Although novaluron has no direct toxicity to adults, the results of this study demonstrate that the delayed lethal activity of this compound reduces hatching of eggs laid by treated adults. Along with the direct ovicidal and larvicidal properties of novaluron, the delayed lethal activity provides an important contribution to the overall control seen in the field.

## Introduction

The codling moth, *Cydia pomonella* (L.) (Lepidoptera: Tortricidae), is one of the most significant pests of temperate pome fruits worldwide (Hoyt et al. 1983; Barnes 1991; Howitt 1993). Historically, *C. pomonella* management tactics have ranged from broad-spectrum insecticides to pheromone-mediated mating disruption (Barnes and Moffitt 1963; Croft and Hoyt 1983; Howell and Maitlen 1983; Riedl et al. 1985; Brunner et al. 2005; Wise et al. 2009; Witzgall et al. 2008). However, due to regulatory changes in the United States under the Food Quality Protection Act (FQPA) of 1996 (Public Law 104–170), registration of many organophosphate and carbamate insecticides have been mitigated, canceled or otherwise altered. With the US EPA (United States Environmental Protection Agency)-mandated phase-out of azinphosmethyl (US EPA 2006) slated for 2012, many new insecticide chemicals have been introduced into USA tree fruit production programs to replace this management tool. However, the FQPA has not been the only incentive to alter older insecticide use patterns in orchards; resistance has also driven the search for replacements in the use against *C. pomonella* and other leafroller species (Varela et al. 1993; Knight et al. 1994; Dunley and Welter 2000; Reuveny and Cohen 2004; Mota-Sanchez et al. 2008; Whalon et al. 2008).

In response to the FQPA, the EPA has established classifications for two new groups: 1) reduced risk and 2) organophosphate-replacements (US EPA 1997). The EPA defines reduced risk insecticides as those that exhibit lower environmental impact and reduced toxicity to humans, mammals and other wildlife, while reducing the risk of groundwater contamination and presumably resistance (US EPA 2006). Of these newer reduced risk and organophosphate-alternative chemicals, the insect growth regulators have demonstrated promise within integrated pest management programs by providing more sustainable attributes, which were outlined by a report issued in 2004 by the United States Department of Agriculture (USDA)/Cooperative State Research, Education, and Extension Service (USDA 2004). Insect growth regulators can be readily implemented into integrated pest management programs due to their reduced potential for environmental and ecological impacts, and thus have played a substantial role in integrated pest management of pome fruits in the United States over the last decade. These compounds are represented by several classes of chemicals that alter the growth and development of the targeted pest.

Benzoylurea insecticides are chitin synthesis inhibitors that are insect growth regulators (Insecticide Resistance Action Committee, Group 15), (IRAC 2010). These compounds interfere with cuticle formation (Mulder and Gijswijt 1973, Ishaaya and Casida 1974; Post et al. 1974), although the exact step in which inhibition occurs remains unclear. Several studies have demonstrated that inhibition of chitin may take place at the polymerization stage of chitin biosynthesis (Hajjar and Casida 1978) or in chitin precursor transport (Nakagawa and Matsumura 1994). Among the variety of benzoylurea compounds, diflubenzuron was one of the first marketed and has been well studied as a chitin synthesis inhibitor. This insecticide has strong larvicidal and ovicidal activity on the target pest, but also demonstrates sublethal activity on *C. pomonella* (Hoying and Riedl 1980; Elliot and Anderson 1982). The term sublethal generally refers to cases in which there are no lethal effects on the life-stage that is in direct contact with the pesticide, but population effects are seen in the subsequent generation as a result of reduced fecundity or egg/larval viability (Wise and Whalon 2009). Depending on the mechanism responsible, these effects may be more accurately called delayed lethal effects.

A new benzoylurea compound, novaluron, was recently developed for use in apple production. Like diflubenzuron, the direct lethal effects of novaluron as an ovicide and larvicide have been the primary basis for its use in *C*. *pomonella* management (Brunner et al 2005). Recent studies examining the delayed lethal effects of novaluron on *C. pomonella* have demonstrated reduced egg hatch after adult exposure (Gökçe et al. 2009). However, the contribution of adult male versus female exposure was not determined. Similar delayed lethal effects of novaluron have been seen in other key pests, but the exact mechanism is unknown. Transovarial movement of the compound from the exposed adult to their eggs and larvae is one mechanism that has been suggested (Cutler et al. 2005; Kostyukosky and Trostanetsky 2006; Wise et al. 2007; Alyokhin et al. 2008). A second suggested explanation for novaluron's activity is the horizontal transfer from contaminated tarsi or scales of previously exposed adults to unexposed eggs, which has been documented with another insect growth regulator (pyriproxyfen) against mosquitoes (Chism and Apperson 2003).

The objectives of this study were to: 1) determine the contribution of adult male versus female exposure to novaluron's delayed lethal effects on hatching of *C. pomonella* eggs and 2) determine if adult mediated horizontal transfer contributes to the effects of novaluron on hatching of eggs.

## Materials and Methods

### Insect material

*C*. *pomonella* pupae were obtained from the Yakima Agricultural Research Laboratory, Wapato, Washington, where the larvae were reared on artificial wheat germ based diet and conditioned in constant light for 24 – 48 hours at 21° C and 60% RH. Male and female pupae were identified according to their abdominal structure and separated into 1-liter plastic containers (Peterson 1965). They were incubated at 21° C, 60% RH and 16:8 L:D photoperiod until adult eclosion. After adult emergence, adult moths were transferred into 1-liter containers and incubated according to experiment type.

### Chemical material

The insect growth regulator novaluron, Rimon® 0.83EC, 9.3% a.i. (Chemtura Corporation, www.chemtura.com), was prepared to the recommended field rate of 30 fl oz per acre (233 ppm). Due to the inability of novaluron to fully homogenize in water, Latron™ B-1956 (Dow AgroSciences LLC, www.dowagro.com) was added at 0.038:1 liter: volume to the novaluron and control solutions. The control treatment was a solution of distilled water and Latron™ B-1956.

### Treatment of one or both sexes

Newly emerged *C*. *pomonella* adults ranging from 1–3 days old were used for all experiments. The female-only experiment consisted of six replicates exposing five unmated females per replicate to novaluron for three days using a modified version of methods used in Gökçe et al. (2009), in which contact and ingestion exposures were combined. The modified version consisted of wax paper that lined the sides and bottom treated with novaluron or the control solution using a handheld sprayer (Lansing Sanitary Supply Inc., www.lssclean.com). In addition to wax paper, a 30 ml plastic cup (SOLO cup company, www.solocup.com) containing 30 ml of treatment or control solution and a protruding cotton dental wick (TIDI® Products, www.tidiproducts.com) was used as a moisture source to prevent dehydration. The control for all experiments consisted of a solution of Latron™ B-1956 and distilled water at a ratio of 0.038:1. After three days female moths were placed into oviposition chambers, which were clean 1-liter containers lined with wax paper and containing five males. Moths were allowed to mate and lay eggs for seven days, after which moths were removed and wax papers were collected. The collected eggs were incubated at 21° C, 60% RH and 16:8 L:D photoperiod for 14 days, after which the number of hatched and non-hatched eggs were counted.

The male-only experiment exposed individuals to novaluron or control for three days, after which moths were placed into the oviposition chambers with five unmated females. The moths were allowed to mate and deposit eggs in the chambers for seven days before they were removed. The eggs were collected incubated at 21° C, 60% RH and 16:8 L:D photoperiod for 14 days, after which the number of hatched and non-hatched eggs was counted. This experiment was replicated six times.

The third experiment treated five females and five males together for three days, after which the same methods were followed as for the single-sex experiments. The collected eggs were incubated at 21° C, 60% RH and 16:8 L:D photoperiod for 14 days, after which the number of hatched and non-hatched eggs was counted. This experiment was replicated six times.

### Horizontal transfer and topical application

Five male and five female *C. pomonella* adults ranging from 1–3 days old were placed into 1-liter containers lined with wax paper for seven days for egg deposition. After the seven day egg laying period the eggs were marked and adults were removed. Separately, five adult unmated females that were previously exposed for three days to novaluron or control by contact and ingestion, as described above, were placed into the container with the marked eggs. Eggs were exposed to the treated females for six days, after which moths were removed and the control and treatment eggs on wax papers were incubated for an additional 14 days to allow for egg hatch. Containers holding the treated moths were visually inspected every 12 hours to verify that adults were contacting previously laid eggs. Contact with eggs was observed as walking over or body contact with eggs. Any new eggs laid by novaluron-exposed females were removed. The experiment was replicated six times and the numbers of hatched and non-hatched eggs were counted.

To test topical application of novaluron onto eggs, five males and five females were placed into 1-liter containers lined with wax paper for seven days to allow for egg deposition. After the seven day period adults were removed and the wax paper with deposited eggs was sprayed with novaluron or control solution until runoff (same concentration as previously stated). Novaluron and control residues were allowed to dry and eggs were incubated for 14 days. The experiment was replicated six times and the numbers of hatched and non-hatched eggs were counted.

**Table 1.**
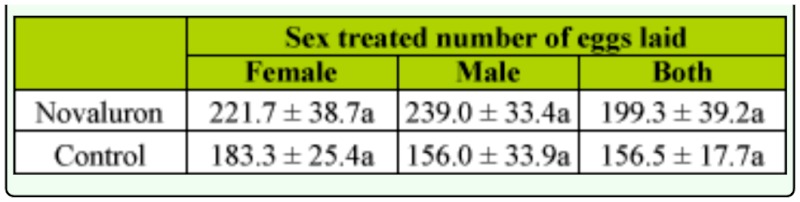
Novaluron treatment of *Cydia pomonella* males (N = 6) or females (N = 6) or both (N = 6) and mean number (±SEM) of eggs laid by codling moth females. Means in columns followed by different letters indicates significant difference (paired t-test, *p* < 0.05).

### Statistical analysis

For all experiments the percentage egg hatch was normalized using arcsine square-root transformation (x′ = arcsine √ x) (Zar, 1999). The transformed data was analyzed by a two-sample t-test (a < 0.05) to determine differences in egg hatch after exposure to the treatment. All statistical analyses were carried out using the SAS statistical program (SAS 2002).

## Results

### Sex treatments

**Figure 1.**
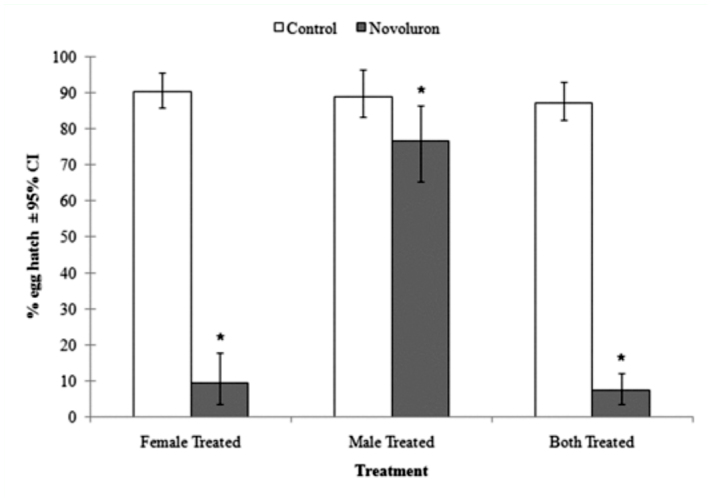
Mean (N = 6) percent hatch of *Cydia pomonella* eggs after treatments of adults (male, female or both sexes) where * indicates that the mean percentage egg hatch (mean ± 95% Cl) is significantly different from the control (two sample t-test, *p* < 0.05). The presented mean egg hatch data were arcsine square-root transformed before analysis and then backtransformed for presentation. High quality figures are available online

The mean percent of hatched eggs laid by treated females was significantly lower than the control (t = 16.31, df = 10, *p* < 0.01) (Figure 1). Although the male-only treatment did not produce the pronounced effect seen in other treatments, the difference between the treatment and the control was still significant (t = 2.60, df = 10, *p* < 0.05). When females and males were both exposed to novaluron, mean percent egg hatch was significantly lower than it was in the control (t = 20.97, df = 10, *p* < 0.01). The results also demonstrated that female-only, male-only, and female-male exposure to novaluron treatments did not affect fecundity (Table 1).

### Horizontal transfer and topical application

**Figure 2.**
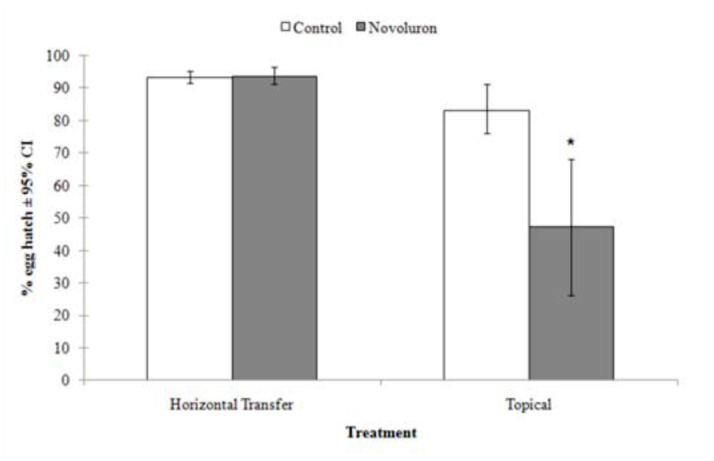
Mean (N = 6) percent hatch of *Cydia pomonella* eggs after different exposure methods where * indicates that the mean percentage egg hatch (mean ± 95% Cl) is significantly different from the control (two sample t-test, *p* < 0.05). The presented mean egg hatch data were arcsine square-root transformed before analysis and then backtransformed for presentation. High quality figures are available online

Eggs exposed to novaluron-treated females showed no difference from the control in the percentage of egg hatch (t = -0.33, df = 10, *p* = 0.7451) (Figure 2). Eggs that were topically treated with novaluron showed significantly lower egg hatch than the control (t = 4.5, df = 10, *p* = 0.01) (Figure 2).

## Discussion

This study demonstrates that the delayed lethal effects of novaluron on the *C. pomonella* can result from both adult male and female exposure, although the impact of male-only exposure on egg hatch is more limited than when adult females are directly exposed. A possible explanation for this is that under male-only exposure novaluron may be transferred to the female during copulation. Moore et al. (1978) demonstrated that the chitin synthesis inhibitor, diflubenzuron, was transferred between sexes in boll weevils and resulted in reduced hatching of eggs laid after treated males were allowed to mate with untreated females.

Novaluron does not appear to affect *C. pomonella* fecundity, regardless of whether males or females are exposed, confirming the results of previously published studies (Gökçe et al. 2009). It has also been demonstrated that exposure to novaluron does not negatively impact the fecundity of female *Leptinotarsa decemlineata* (Alyokin et al. 2008, 2009) or *Tribolium castaneum* (Kostyukovsky and Trostanetsky 2006).

The lack of significant reduction in egg hatching in the horizontal transfer experiment suggests that transfer of novaluron by contaminated females onto previously laid eggs is not a valid explanation for the delayed lethal effects previously claimed for this compound. However, other exposure methods such as direct/topical contact should be explored before ruling out horizontal transfer. The results from the topical application confirm what has already been published on the direct ovicidal effects of novaluron on *C. pomonella* (Brunner et al. 2005). The results presented in this study and by Gökçe et al. (2009), demonstrate that the observed reduction in egg hatch is likely related to the transfer of the compound within the female, as opposed to superficial contact of exposed females to eggs.

The exact mechanism or mechanisms responsible for the observed delayed lethal effects is yet to be determined. There is evidence for a physiological mechanism whereby exposure to diflubenzuron resulted in compromised reproductive organs (Soltani and Soltani-Mazouni 1992). Transovarial transfer to eggs as a mechanism has gained evidence from several researchers using diflubenzuron or novaluron (Elek 1998; Medina et al. 2002; Kostyukovsky and Trostanetsky 2006; Trostanetsky and Kostyukosky 2008). Medina et al. (2002) demonstrated presence of benzylurea in the ovaries and eggs of *Chysoperla carnea* using radio-labeled diflubenzuron after adults were exposed to the compound. Numerous observations were made in our study of eggs in the novaluron treatments that failed to eclose while in the black-head stage, which provides further support for the transovarial mechanism of activity. If the mechanism is transovarial in nature then research is still needed to determine the morphological pathway the compound takes from the adult moth to the eggs. Research conducted by Alyokin et al. (2009) showed that Colorado potato beetles treated continuously with novaluron-treated foliage resulted in failure of egg hatch, but egg hatch was restored after several days exposure to untreated foliage.

In pome fruit integrated pest management programs, novaluron use is primarily based on its ovicidal and larvacidal properties throughout the growing season. However, the contribution of novaluron's delayed lethal activity needs to be considered when applying field treatments. With the imminent phase-out of azinphosmethyl, the newer classes of insecticides being marketed must be well understood and their patterns of use optimized. Incorporation of novaluron into *C. pomonella* mating disruption programs may result in enhanced seasonal control of this key pest. Although mating disruption has been successful in low populations, the migration of mated females into mating disruption areas and areas with high populations require chemical applications to prevent fruit damage (Brunner et al. 2002, Witzgall et al. 2008). The addition of the lethal and delayed lethal effects of novaluron can complement the adult effects of mating disruption in an integrated pest management system.

More research is needed to fully understand the mechanisms responsible for its effects on reduced hatching of eggs seen in this study and in Gökçe et al. (2009). Future research is needed to determine how transovarial novaluron affects egg hatch, and if reduced egg hatch is measureable under field conditions.

## Abbreviatons:

**FQPA**,: Food Quality Protection Act;
**US EPA**,: United States Environmental Protection Agency
